# Characteristics, pathophysiological mechanisms, and ablation outcomes of patients with de novo left atrial flutter compared with patients with postablation left atrial flutter

**DOI:** 10.1016/j.hroo.2026.03.027

**Published:** 2026-03-27

**Authors:** Valon Spahiu, Thomas Kueffer, Ruben Fuentes Artiles, Berat Gerguri, Salik ur Rehman Iqbal, Antonio Madaffari, Gregor Thalmann, Claudia Herrera, Boldizsar Kovacs, Nikola Kozhuharov, Corinne Jufer, Helge Servatius, Andreas Haeberlin, Fabian Noti, Hildegard Tanner, Laurent Roten, Tobias Reichlin

**Affiliations:** 1 Department of Cardiology, Inselspital – University Hospital Bern, University of Bern, Bern, Switzerland; 2 SITEM Center for Translational Medicine and Biomedical Entrepreneurship, University of Bern, Bern, Switzerland; 3 Department of Internal Medicine, Salem Hospital, Bern, Switzerland; 4 Unità operativa cardiologia indirizzo elettrofisiologia, Ospedale Santa Maria del Carmine di Rovereto, Trentino-Alto Adige, Italy

**Keywords:** Left atrial flutter, Pulsed field ablation, Radiofrequency ablation, Electrophysiology, Pulmonary vein isolation, Atrial arrhythmia, 3D mapping

## Abstract

**Background:**

Left atrial flutter (LAF) most commonly arises in patients with previous left atrial ablation or surgery. Rarely, it can present as a de novo arrhythmia without previous interventions.

**Objective:**

We aimed to compare patient characteristics, pathophysiological mechanisms, and ablation outcomes of patients with de novo LAF with those of patients with postablation LAF.

**Methods:**

Patients undergoing LAF ablation between January 2021 and June 2023 were included. Baseline data, arrhythmia mechanisms, and ablation targets were collected. Patients were followed with 7-day Holter electrocardiograms at 3, 6, and 12 months. The primary endpoint was arrhythmia recurrence after a 90-day blanking period.

**Results:**

A total of 147 patients with LAF were analyzed (median age 71 years; 65% men). Of those, 32 (22%) presented with de novo LAF, and 115 (78%) presented with postablation LAF. Patients in the de novo group were older (74 years vs 70 years; *P* = .037), were more often women (50% vs 30%; *P* = .058), and had a lower body mass index (26.0 vs 27.1; *P* = .025). In 28% of patients in the de novo group, atrial fibrillation had not been diagnosed before the procedure. The dominant arrhythmia mechanisms in the de novo group were anteroseptal localized reentry (44%), followed by perimitral flutter (31%) and roof-dependent flutter (16%). Freedom from any atrial arrhythmia at 12 months was 46% (95% confidence interval 30–69) in the de novo and 50% (95% confidence interval 41–62) in the postablation group (*P* = .39).

**Conclusion:**

Patients with de novo LAF are older and leaner than patients with postablation LAF, and the predominant arrhythmia mechanism is anteroseptal localized reentry. Arrhythmia-free survival is similar in both groups.

## Graphical abstract

**Figure:**
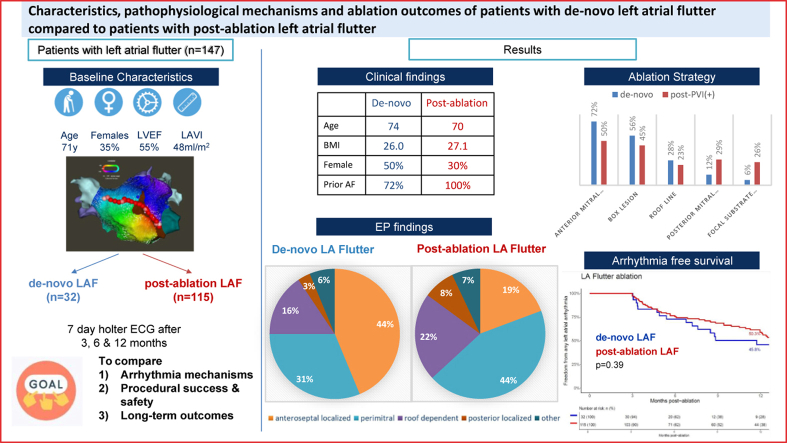

**Key Findings**

▪ De novo patients were older, had a lower body mass index, and were more often women. In nearly one-third of these patients, no atrial fibrillation had been documented before presentation.
▪ Differences in arrhythmia mechanisms were observed between the groups. Anteroseptal localized reentry was the most common arrhythmia mechanism in the de novo group.
▪ Ablation procedures were generally safe, with no significant difference in complication rates between the 2 groups.
▪ Catheter ablation demonstrated comparable effectiveness in both cohorts, with no significant difference in arrhythmia-free survival.

## Introduction

Left atrial flutter (LAF) is a well-recognized tachycardia after ablation of atrial fibrillation (AF). These arrhythmias have been well described, with a reported incidence in post–pulmonary vein isolation (PVI) patients ranging from 2.9% to as high as 31%.^1^, ^2^, ^3^, ^4^, ^5^, ^6^ The wide variability of LAF incidence reflects differences in study populations, ablation targets, rhythm monitoring strategies during follow-up, and follow-up duration. The most common type of LAF after AF ablation is perimitral flutter, followed by roof-dependent atrial flutters.^5^^,^^7^ Mapping and subsequent ablation of these organized left atrial (LA) arrhythmias are usually possible, with reported 3-year success rates between 38% and 73%.^5^^,^^6^

In contrast, de novo LAF occurs in patients without previous ablation or cardiac surgery, representing a rare form of organized left atrial tachycardia (AT). To date, only limited data are available on the incidence, mechanisms, and outcome of de novo LAF.^8^^,^^9^

Our study aimed to compare patient characteristics, pathophysiological mechanisms, and arrhythmia-free outcomes of patients with de novo LAF with those of patients with postablation LAF in a large series of consecutive patients undergoing ablation for LAF.

## Methods

### Study population

This study is a prospective analysis of consecutive patients undergoing catheter ablation for documented LAF in a large tertiary referral center between January 2021 and June 2023. Patients were enrolled through an institutional electrophysiology ablation registry that captures all catheter ablation procedures performed for atrial arrhythmias during routine clinical care. A written informed consent was obtained from patients for participation in the study and publication of the clinical data. The registry received approval from the local ethics committee (KEK PB_2018_00226), and the study adhered to the principles outlined in the Declaration of Helsinki. The authors had unrestricted access to the data and vouch for its accuracy and reliability.

LAF was defined as a sustained AT originating from the LA with 12-lead electrocardiographic (ECG) documentation and origin confirmed during 3-dimensional electroanatomic mapping (3D-EAM) during the index procedure. Consecutive patients included without other inclusion or exclusion criteria and were categorized into 2 groups: de novo LAF, defined as LAF without previous LA ablation or cardiac surgery, and postablation LAF, which was defined as LAF after at least 1 previous LA ablation, including PVI or PVI with additional extrapulmonary vein ablation targets (PVI+). Patient demographics, baseline characteristics, medication use, and procedural data were collected prospectively and extracted from the institutional electronic patient records. Arrhythmia mechanisms and procedural strategies were recorded in detail in all procedural reports.

AF was classified as paroxysmal if episodes terminated spontaneously within 7 days or as persistent if episodes lasted longer than 7 days or required cardioversion. AF duration was defined as the time from the first ECG documentation to the index LAF ablation. LA volume index (LAVI) and left ventricular ejection fraction were obtained from the most recent transthoracic echocardiogram, typically performed on the day before the procedure.

### Preprocedural examinations and periprocedural management

All patients underwent preprocedural transesophageal echocardiography and/or cardiac computed tomography angiography to exclude intracardiac thrombi and to assess LA anatomy. Procedures were predominantly performed under deep sedation guided by a physician-led, nurse-administered protocol using midazolam, fentanyl, and propofol or under anesthesia in selected high-risk patients.^10^^,^^11^ LA access was obtained by fluoroscopy-guided transseptal puncture or through a patent foramen ovale, as applicable.^12^ Intraprocedural anticoagulation was achieved with heparin at the time of venous access to maintain an activated clotting time of >350 seconds.

### Mapping of the arrhythmia circuits

A high-density 3D-EAM system was used in all patients (Biosense Webster, Johnson & Johnson, New Brunswick, NJ).^13^ A dedicated multipolar mapping catheter (Pentaray or Octaray catheter, Biosense Webster, Irvine, CA) was used to delineate the LA anatomy and identify the arrhythmogenic circuits by means of high-density activation, voltage, and entrainment mapping during ongoing LAF.

Arrhythmia mechanisms were classified based on high-density activation mapping, voltage mapping, and entrainment maneuvers when feasible, according to the predominant tachycardia circuit. Macroreentrant tachycardias were defined by continuous atrial activation covering more than 90% of the tachycardia cycle length and consistent entrainment responses from various sites within the circuit. Given the overlapping electroanatomic features of focal and microreentrant tachycardias on high-density mapping and their similar ablation strategy, these mechanisms were analyzed together as localized reentry. Tachycardias were subsequently subcategorized according to the predominant anatomic location of the critical circuit or earliest activation site as anteroseptal tachycardias (focal or microreentrant circuits localized in the anteroseptal region of the LA), perimitral macroreentry (large reentrant circuits revolving around the mitral valve annulus), roof-dependent macroreentry (large reentrant circuits involving the roof of the LA), posterior wall tachycardias (focal or microreentrant circuits localized to the posterior wall), or as other localized reentry (focal or microreentrant circuits involving other areas of the LA). If the arrhythmia mechanism could not be determined owing to the continuous change of the cycle length and/or activation, the arrhythmia was classified as unmappable. In patients with previous PVI, the pulmonary veins were assessed for conduction recovery.

### Ablation strategy

The ablation strategy was consistent across both the de novo and postablation groups, targeting the arrhythmia mechanism using predefined linear ablation targets. Radiofrequency ablation (RFA), pulsed field ablation (PFA), or a combination of both energy sources was used at the operator’s discretion, depending on their preference and the specific anatomic and procedural circumstances. For RFA, an irrigated contact force–sensing ablation catheter was used (Smarttouch SF, Biosense Webster). For PFA, a multipolar pentaspline PFA catheter was used (Farawave, Boston Scientific, Menlo Park, CA). To ensure precise catheter tracking and positioning for accurate and effective ablation, the PFA catheter was visualized in the 3D-EAM system, as previously described.^13^

To effectively address the identified arrhythmia circuits, the critical isthmuses identified by a combination of activation, entrainment, and voltage mapping were targeted for ablation and connected to unexcitable structures by means of linear ablation. For ablation of the posterior mitral isthmus, epicardial ablation from the coronary sinus and/or ethanol ablation of the vein of Marshall were performed as adjunctive strategies to endocardial ablation, if required.^14^^,^^15^ The procedural end point was a bidirectional conduction block across the ablation lines.

In the de novo LAF group, PVI was performed as part of the standard lesion set in most patients. In the postablation group, reisolation of reconnected pulmonary veins was performed. The end point of PVI in both groups was verification of entrance and exit block at the end of the procedure.

### Follow-up

Antiarrhythmic drugs (class I and III) were stopped after the procedure or at day 90 after ablation. Follow-up included scheduled outpatient visits with 7-day Holter monitoring at 3, 6, and 12 months after the index procedure. Recurrent symptoms prompted additional symptom-triggered 12-lead ECGs and interim Holter monitoring at the discretion of the treating physician.

The primary end point was the recurrence of any atrial arrhythmia after a single procedure, lasting more than 30 seconds, and after a 90-day blanking period. The safety outcome was a composite of in-hospital major adverse events (vascular injury requiring intervention, stroke/transient ischemic attack, cardiac tamponade, cardiac decompensation, and bradycardia requiring intervention).

### Statistical analysis

Continuous variables are presented as mean ± standard deviation or as median and interquartile range (IQR), as appropriate. The primary end point was estimated using the Kaplan-Meier method, and survival curves were compared using the log-rank test. Comparisons between independent groups were made using the χ^2^ or Fisher’s exact test for categorical variables and using the Mann-Whitney U test for continuous variables. To account for baseline imbalances between groups, a multivariable Cox proportional hazards model was constructed with prespecified covariates including postablation, sex, age, body mass index (BMI), LAVI, and AF history.

The multiple imputation by chained equations method was used to account for missing data.

All tests were 2 sided, and *P* < .05 was considered statistically significant. Statistical analyses were performed using R 4.3.2 (R Core Team, Vienna, Austria)

## Results

### Patient characteristics

A total of 147 patients undergoing ablation of LAF were included in the study. The median age was 71 years (IQR 65–75 years), and 65% of the cohort were men. Of those, 32 patients (22%) presented with de novo LAF, and 115 patients (78%) presented with postablation LAF.

Baseline characteristics of the patients are presented in Table 1. Patients in the de novo group were older (74 years vs 70 years; *P* = .037), were more often women (50% vs 30%; *P* = .058), and had a lower BMI (26.0 vs 27.1; *P* = .025). In 28% of patients in the de novo group, no AF had been diagnosed before. In the postablation group, patients had undergone a mean of 1.6 previous ablation procedures, including 54 with PVI only and 61 with PVI and additional extrapulmonary vein ablations (PVI+). Among the 7 postablation patients (6% of the cohort) (Table 1) without a history of AF, 4 had undergone ablation for LA focal tachycardia, and 3 had undergone ablation for de novo LAF during their previous procedures. The number of previous ablation lines per patient ranged from 1 to 2, with a total of 10 ablation lines performed.

**Table 1:** Characteristics of patients at baseline, procedure, and outcome data

Variables	Total (N = 147)	De novo (n = 32)	Postablation (n = 115)	*P* value
Age, y	71.4 (65.3–75.3)	73.6 (69.0–78.1)	70.0 (64.5–75.2)	.037
Male sex	96 (65.3)	16 (50.0)	80 (69.6)	.058
Body mass index, kg/m^2^	27.0 (24.0–31.0)	26.0 (22.5–28.2)	27.1 (24.5–31.6)	.025
CHA_2_DS_2_-VASc score	3.0 (2.0–4.0)	3.0 (2.0–4.0)	3.0 (2.0–4.0)	.211
Concomitant clinical conditions				
Hypertension	101 (68.7)	20 (62.5)	81 (70.4)	.397
Coronary artery disease	34 (23.1)	8 (25.0)	26 (22.6)	.814
Diabetes	23 (15.6)	3 (9.4)	20 (17.4)	.410
Previous stroke or TIA	14 (9.5)	5 (15.6)	9 (7.8)	.187
Atrial fibrillation type				.004
None	16 (10.9)	9 (28.1)	7 (6.1)	
Paroxysmal	60 (40.8)	10 (31.2)	50 (43.5)	
Persistent	71 (48.3)	13 (40.6)	58 (50.4)	
Number of years since the first diagnosis of atrial fibrillation	5.3 (2.4–8.6)	2.5 (0.3–4.5)	5.9 (3.0–9.1)	<.001
Previous left atrial ablations				<.001
0	32 (21.8)	32 (100.0)	0 (0.0)	
1	62 (42.2)	0 (0.0)	62 (53.9)	
2	38 (25.9)	0 (0.0)	38 (33.0)	
3	15 (10.2)	0 (0.0)	15 (13.0)	
Total number of previous left atrial lines	118	0	118	<.001
0	87 (59.2)	32.0 (100%)	55 (47.8)	
1	18 (12.9)		18 (16.5)	
2	26 (17.7)		26 (22.6)	
3	13 (8.8)		13 (11.3)	
4	2 (1.4)		2 (1.7)	
Median number of previous left atrial lines	0.00 (0.00–2.00)	0.00 (0.00–0.00)	1.00 (0.00–2.00)	<.001
Mean number of previous left atrial lines	0.80 (1.10)	0.00 (0.00)	1.02 (1.15)	<.001
Antiarrhythmic drug				
Class I	7 (4.8)	1 (3.1)	6 (5.2)	1.000
Class II	120 (81.6)	24 (75.0)	96 (83.5)	.305
Class III	27 (18.4)	9 (28.1)	18 (15.7)	.124
Echocardiographic data				
Left atrial volume index, mL/m^2^	48.0 (39.0–64.5)	51.0 (43.0–67.0)	47.0 (39.0–60.0)	.361
Left ventricular ejection fraction, %	55.0 (45.0–58.0)	53.5 (40.0–60.0)	55.0 (45.0–55.0)	.798

Numbers are n (%) or median (interquartile range) as appropriate. Categorical variables (eg, sex, comorbidities, and antiarrhythmic drug use) are shown as counts with percentages, whereas continuous variables (eg, age, body mass index, left atrial volume index, left ventricular ejection fraction, and atrial fibrillation duration) are reported as medians with interquartile ranges.

TIA = transient ischemic attack.

### Procedural characteristics

Procedural, arrhythmia, ablation, and safety characteristics are presented in Table 2. Median procedure times were comparable between the groups (IQR 134–210 in the de novo group [186 minutes] vs IQR 127–194 in the postablation group [160 minutes]; *P* = .10). Fluoroscopy time also was similar between the groups (IQR 13–27 [20 minutes] vs IQR 9–27 [18 minutes]; *P* = .48).

**Table 2:** Procedural and safety data

Variables	Total (N = 147)	De novo(n = 32)	Postablation (n = 115)	*P* value
Procedure duration, min	165.0 (129.5–200.0)	185.5 (134.2–209.5)	160.0 (126.5–193.5)	.096
Fluoroscopy time, min	18.0 (9.3–27.0)	20.0 (12.5–27.0)	17.6 (9.0–27.0)	.477
Ablation technology				.113
Pulsed field	39 (26.5)	12 (37.5)	27 (23.5)	
Radiofrequency	74 (50.3)	11 (34.4)	63 (54.8)	
Pulsed field and radiofrequency	34 (23.1)	9 (28.1)	25 (21.7)	
Ethanol ablation	8 (5.4)	2 (6.3)	6 (5.2)	1.000
Number of linear lesions	1.0 (1.0–2.0)	2.0 (1.0–2.0)	1.0 (1.0–2.0)	.279
Lesion set				
Pulmonary vein isolation	84 (57.1)	30 (93.8)	54 (47.0)	<.001
Roof line	36 (24.5)	9 (28.1)	27 (23.5)	.758
Box lesion	70 (47.6)	18 (56.2)	52 (45.2)	.365
Anterior mitral isthmus line	81 (55.1)	23 (71.9)	58 (50.4)	.050
Posterior mitral isthmus line	37 (25.2)	4 (12.5)	33 (28.7)	.102
Focal substrate modification	32 (21.8)	2 (6.2)	30 (26.1)	.031
Complications	13 (8.8)	2 (6.2)	11 (9.6)	.816
Minor	9 (6.1)	2 (6.2)	7 (6.1)	1.000
Major	4 (2.7)	0 (0.0)	4 (3.5)	.649

Numbers are n (%) or median (interquartile range) as appropriate.

### Arrhythmia mechanisms

**Figure 1:**
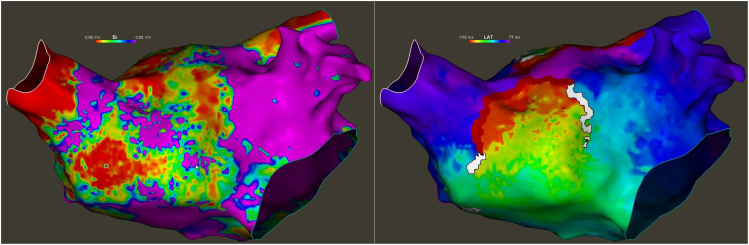
Electroanatomic activation and voltage maps of a patient with de novo left atrial flutter. *Left:* A high-density bipolar voltage map showing a septal scar. *Right:* An activation map of a de novo left atrial flutter demonstrating the propagation of the activation wavefront of a localized reentry in the septal wall.

**Figure 2:**
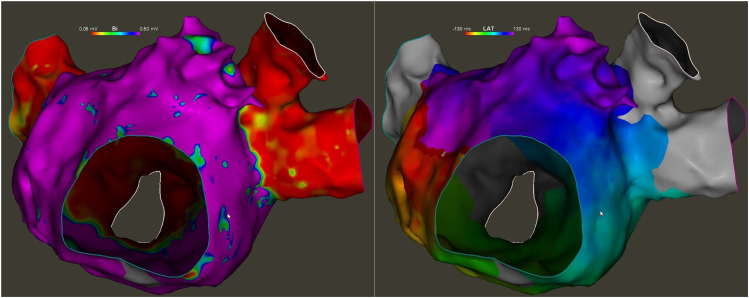
Electroanatomic activation and voltage maps of a patient with postablation left atrial flutter. *Left:* A high-density bipolar voltage map of a patient with macroreentrant left atrial flutter after previous pulmonary vein isolation. *Right:* The activation map of the same patient demonstrates a perimitral flutter circuit.

In the de novo group, anteroseptal localized reentry (Figure 1) was the most common arrhythmia mechanism (14 of 32; 44%) identified by high-density 3D-EAM. Perimitral macroreentry accounted for 31% (10 of 32), roof-dependent flutter for 16% (5 of 32), and posterior wall localized reentry for 3% (1 of 32), whereas unmappable cases represented 6% (2 of 32). In the postablation group, perimitral macroreentry (Figure 2) was the dominant mechanism (50 of 115; 44%). Anteroseptal localized reentry was observed in 19% of cases (22 of 115), roof-dependent flutter in 22% (25 of 115), posterior wall localized reentry in 8% (9 of 115), and other localized reentries in 4% (4 of 115), and unmappable cases constituted 4% (5 of 115).

One-third of patients had more than 1 AT mechanism (49 of 147; 33.3%), with similar frequency in the de novo and postablation cohorts (28.1% vs 34.8%; *P* = .47). Of these, dual-loop reentry was the most common phenomenon present in 90% of patients (Table 3) and numerically more frequent in the postablation group (21.9% vs 32.2%; *P* = .26).

**Table 3:** Tachycardia mechanisms

Variables	Total (N = 147)	De novo (n = 32)	Postablation (n = 115)
Tachycardia mechanism			
Localized reentry	50 (35.7)	15 (50.0)	35 (31.8)
Anteroseptal	36 (24.5)	14 (43.8)	22 (19.1)
Posterior wall	10 (6.8)	1 (3.1)	9 (7.8)
Other	4 (2.7)	0 (0.0)	4 (3.5)
Macroreentry	90 (64.3)	15 (50.0)	75 (68.2)
Perimitral	60 (40.8)	10 (31.3)	50 (43.5)
Roof dependent	30 (20.4)	5.0 (15.6)	25 (21.7)
Unmappable	7 (4.8)	2.0 (6.3)	5 (4.3)
Complex tachycardias			
Multiple left atrial tachycardia	49 (33.3)	9 (28.1)	40 (34.8)
Dual-loop tachycardia	44 (29.9)	7 (21.9)	37 (32.2)
Tachycardia termination			
All tachycardias terminated	131 (89.1)	28 (87.5)	103 (89.6)
Reason for no termination	16 (10.9)	4 (12.5)	12 (10.4)
Rapidly changing tachycardias or degeneration into AF	11 (7.5)	2 (6.3)	9 (7.8)
No bidirectional block	5 (3.4)	2 (6.3)	3 (2.6)

Numbers are n (%).

AF = atrial fibrillation.

### Arrhythmia mechanisms, previous ablation lesions, and areas of low voltage

The relationship among circuit mechanism, voltage abnormality, and previous ablation lesion sets was examined in the postablation group (Supplemental Table 1). There was a concordance between the underlying substrate and the arrhythmia mechanism. Anteroseptal localized circuits were significantly associated with a previous anteroseptal ablation line (28% vs 2%; *P* < .001), and similar findings were found in posterior localized reentry circuits, which were more common in areas of previous posterior wall ablation (17% vs 1%; *P* = .004). Perimitral circuits were the most frequent mechanisms across all scar types and ablation line groups.

In the de novo group, anteroseptal circuits were predominantly associated with anteroseptal voltage abnormality, but the relatively low number of patients makes direct comparison challenging (Supplemental Table 2). Overall, anteroseptal voltage abnormality was the most common finding, present in 86% of anteroseptal localized reentry (31 of 36), 57% of perimitral circuits (34 of 60), 47% of roof-dependent flutter (14 of 30), and 100% of unmappable tachycardias (7 of 7) (Supplemental Table 3).

### Ablation strategy and acute outcomes

RFA alone was used in 50% of cases, PFA alone in 27%, and a combination of both modalities in 23%. Ethanol ablation was used in 8 patients.

Patients in the de novo group more frequently underwent anterior mitral isthmus line ablation (from the right superior pulmonary vein to the mitral anulus) than the postablation group (72% vs 50%; *P* = .04), whereas focal substrate modification was performed less often (6% vs 26%; *P* = .02). PVI was performed in 94% of cases in the de novo group and redo PVI in 47% in the postablation group. In 2 patients (6%) from the de novo group, PVI was not performed owing to a microreentry mechanism of the tachycardia not requiring linear ablation and the absence of an AF diagnosis at the time of ablation. Acute bidirectional block was achieved and documented for 97% of linear ablations in both groups. For posterior wall isolation, complete acute isolation was achieved in 100% of cases using both technologies or the combination of them. During ablation, 23 patients (16%) demonstrated a transition to a second LAF circuit, including 6 patients (19%) in the de novo group and 17 patients (15%) in the postablation group.

Acute termination of LAF after ablation was achieved in 89% of patients. In the remaining 16 patients (11%), tachycardia required termination by direct current cardioversion or overdrive pacing owing to cycle-length instability, degeneration into AF, or failure to achieve acute bidirectional block despite extensive ablation (Table 3).

### Procedural safety

Adverse events were observed in 4 patients overall (2.7% of all patients), with a rate of 3.5% in the postablation group and 0% in the de novo group (*P* = .65). These included 1 case of pericardial tamponade requiring pericardiocentesis and 3 cases of subsequent postprocedural heart failure exacerbation requiring a prolonged hospital stay. There were no cases of stroke, esophageal injury, phrenic nerve palsy, or access site bleeding requiring intervention.

### Long-term outcomes after ablation of de novo and postablation LAF

**Figure 3:**
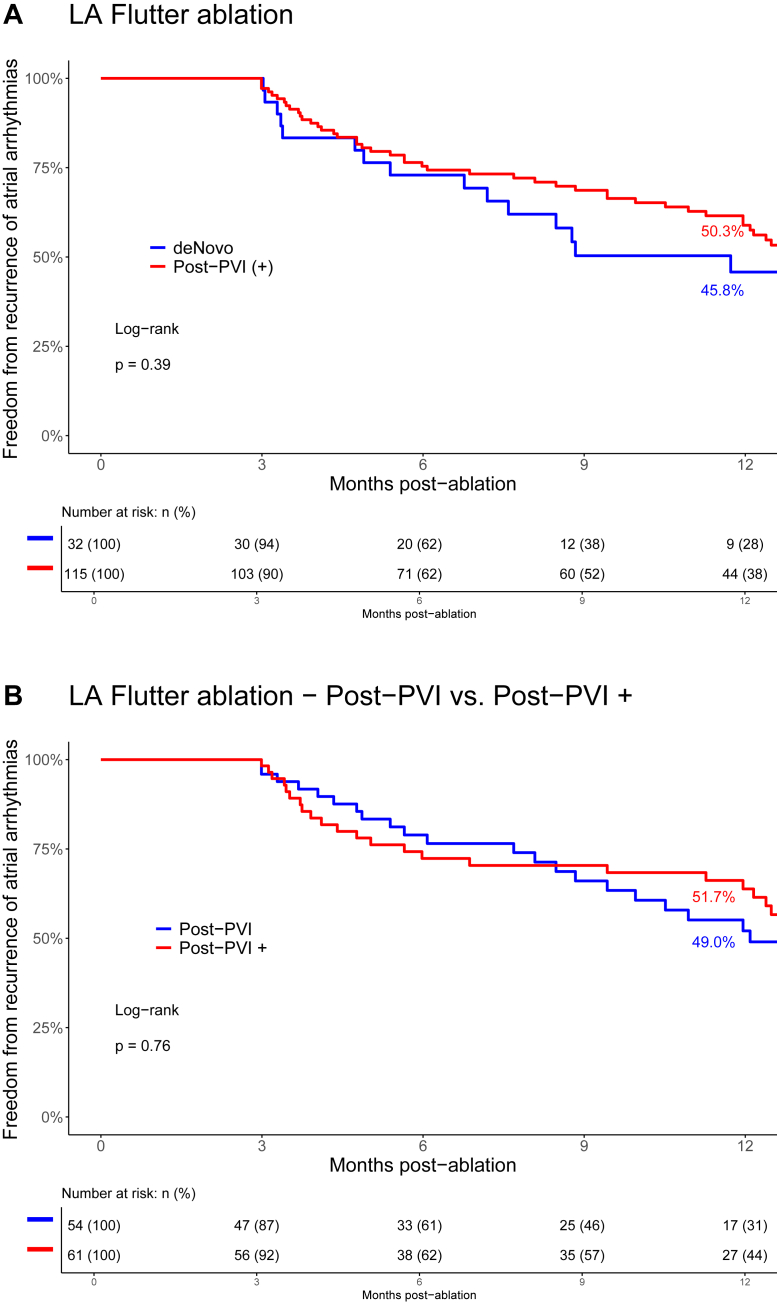
Recurrence of atrial arrhythmias after ablation of LAF. Kaplan-Meier curves displaying freedom from any atrial arrhythmia after LAF ablation. **A:** A de novo LAF vs postablation LAF. **B:** Patients with postablation LAF with previous PVI alone vs additional LA ablation beyond PVI. LA = left atrial; LAF = left atrial flutter; PVI = pulmonary vein isolation.

Patients were followed for a median duration of 16 months (IQR 10–25 months). At 12-month follow-up, freedom from atrial arrhythmias lasting >30 seconds was observed in 46% (95% confidence interval 30–69) in the de novo group and 50% (95% confidence interval 41–62) in the postablation group (*P* = .39) (Figure 3A). No difference was observed in the postablation group between patients after PVI only compared with patients after PVI+ (Figure 3B). A sensitivity analysis was performed comparing patients ablated with RFA only and PFA only, showing no difference in arrhythmia-free outcome at 12 months (Supplemental Figures 1 and 2). The pattern of recurrence was different in the 2 groups: organized ATs were the dominant recurring arrhythmia in patients with de novo LAF (13 of 15; 87%), whereas recurring AF was rare (2 of 15; 13%). In patients with postablation LAF, this was evenly split (23 of 46; 50% each for organized ATs and AF).

A multivariate Cox proportional hazards regression analysis was performed to assess factors associated with the recurrence of atrial arrhythmias, including postablation, sex, age, BMI, LAVI, and AF history. None of these factors were found to be significantly associated with recurrence, as shown by the hazard ratios and corresponding *P* values (Table 4).

**Table 4:** Multivariable analysis: Factors associated with recurrence of atrial arrhythmias (N = 147)

Variables	HR	95% CI	*P* value
Postablation	0.70	0.36–1.33	.267
Male sex	1.30	0.72–2.35	.373
Age, y	1.00	0.97–1.04	.897
Body mass index, kg/m^2^	1.03	0.97–1.09	.295
Left atrial volume index, mL/m^2^	0.99	0.98–1.01	.411
Previous AF	0.83	0.32–2.11	.687

AF = atrial fibrillation; CI = confidence interval; HR = hazard ratio.

Repeat ablation was performed in 10 patients (32%) in the de novo group and in 32 patients (28%) in the postablation group after a median of 30.2 months (IQR 16.6–40.7). In 34 of those patients (83%), a different arrhythmia mechanism compared with the index procedure was found. Only in 7 (17%) was the same arrhythmia mechanism found. All of those initially had perimitral flutter with recurrence of conduction across the mitral isthmus line (4 patients with an initial anterior mitral isthmus line [from the right superior pulmonary vein to the anterior mitral annulus] and 3 patients with a posterior mitral isthmus line [from the left inferior pulmonary vein to the posterior mitral annulus]).

The recurrence mechanisms were heterogeneous in 42 patients undergoing a redo procedure (Supplemental Table 4). A tachycardia mechanism arising from the anatomically corresponding scar region during the index procedure was identified in only 5 patients (11.9%). AF was the most common indication for a redo procedure, occurring in 19 patients (45.2%), and in 12 patients (28.6%), the tachycardia was unmappable.

Of the 8 patients (5%) who underwent ethanol ablation of the vein of Marshall at the index procedure, 3 (37.5%) experienced arrhythmia recurrence during follow-up. Notably, none of these recurrences were caused by perimitral flutter (2 anteroseptal localized atrial flutters and 1 roof-dependent flutter), and bidirectional mitral isthmus block was confirmed in all redo procedures.

In addition to redo procedures for sustained symptomatic recurrence, arrhythmia-related hospitalizations that did not result in repeat ablation procedures were also analyzed, occurring in 3 patients (9%) in the de novo group and 2 patients (2%) in the postablation group.

## Discussion

This prospective study of 147 consecutive patients undergoing ablation of LAF represents 1 of the most comprehensive collections of patients with de novo LAF without previous LA ablations or surgery. We report 4 major findings:

First, de novo group patients were older, had a lower BMI, and were more often women. In almost one-third of the patients, no AF had been documented before. Second, differences in arrhythmia mechanisms were observed between the groups, with anteroseptal localized reentry being the most common arrhythmia mechanism in the de novo group. Third, ablation procedures were generally safe with no differences in the complication rate between the 2 groups. Finally, catheter ablation was similarly effective for both cohorts without a significant difference in arrhythmia-free survival.

Our findings corroborate and expand previous studies: Zaidi et al^9^ reported on 58 cases of de novo LAF and concluded that the most common mechanism was localized flutter in the anterior or anteroseptal LA (19 of 58 cases), followed by perimitral macroreentry in 12 of 58 patients. Yakabe et al^8^ reported on 22 patients with de novo atypical flutter. In their study, they also included patients with right atrial non–cavotricuspid isthmus flutters. Their main finding was that patients with atypical flutter had distinctive electrophysiological characteristics suggestive of atrial cardiomyopathies, with spontaneous atrial scar observed in all 22 cases. The most frequent location of scarring in the LA was the anterior wall (8 cases). The recurrence rate of all atypical flutter cases at 3.5 years of follow-up reported by Yakabe et al^8^ was 63.6%, whereas Zaidi et al^9^ reported a recurrence rate of 75% at a median follow-up of 3.2 years for all atypical flutter cases.

As a unifying finding, all 3 studies reported a noteworthy predilection for anterior/anteroseptal localized reentry in patients with de novo LAF. The pathophysiological etiology of the septal fibrosis in these patients is unclear. Emerging data suggest that LA voltage abnormalities may be influenced not only by intrinsic fibrotic remodeling but also by anatomic relationships with adjacent structures such as the aorta or pulmonary artery. Previous studies, including work by Zhu et al,^16^ have described associations among regional low-voltage areas, spontaneous atrial scarring, and macroreentrant AT circuits, supporting the concept that anatomic and structural factors may contribute to the arrhythmogenic substrate underlying LAF. The clinical phenotype with a higher proportion of women, older age, and lower BMI is notable. It might indicate a potential role of diastolic dysfunction. Detailed echocardiographic data and natriuretic peptide levels to quantify left ventricular filling pressures were unfortunately not available in our study to further explore this hypothesis.^17^

By comparing patients with de novo LAF and postablation LAF, our study adds important data to the previously discussed single-arm studies.^8^^,^^9^ Notably, we observed comparable long-term outcomes after ablation in both groups. This has important clinical implications, given that it indicates that all patients with LAF regardless of the underlying etiology can be managed successfully using the same ablation strategy. Furthermore, outcomes were comparable between RFA and first-generation pentaspline PFA in our study. Meanwhile, a second-generation PFA system using a dual-energy large area focal lattice-tip catheter with full 3-dimensional integration and capable of delivering both RFA and PFA has shown encouraging results for linear ablation and might be particularly suitable for the treatment of patients with LAF.^18^

In our de novo cohort, of the 9 patients with truly isolated LAF without documented AF, only 2 did not undergo PVI, which precludes any firm conclusions regarding the necessity of PVI in this specific subgroup.

Finally, in patients undergoing repeat procedures, a different arrhythmia mechanism compared with the index procedure was found in the vast majority of patients. This most likely indicates an evolution of the substrate and the development of new reentry circuits, but could also relate to incomplete ablation of arrhythmia substrate that was not involved in the initial arrhythmia mechanism.

### Limitations

Our study has several limitations that should be considered when interpreting our findings. First, this was a single-center study, and replication of our findings from other centers will be insightful. Second, some of the procedures were performed with RFA, some with PFA, and some with a combination of both. Measures to minimize bias and confounding included consistent procedural standards and follow-up protocols across all patients, and a sensitivity analysis restricted to either ablation modality. Third, systematic cycle-length data were not collected for all tachycardias, which limited our ability to perform more detailed mechanistic comparisons between groups. Nevertheless, residual confounding affecting our results cannot be completely excluded.

## Conclusion

Patients with de novo LAF differ from those with postablation LAF in that the arrhythmia mechanism in de novo LAF is predominantly anteroseptal localized reentry, whereas postablation LAF often involves macroreentrant circuits related to previous ablation sites. Despite these differences in arrhythmia substrate, freedom from atrial arrhythmia at 12-month follow-up was comparable in both groups and regardless of the ablation technology used.

## Declaration of generative AI and AI-assisted technologies in the writing process

The authors used ChatGPT-4o to enhance the clarity and readability of the manuscript. All content generated was reviewed and edited by the authors, who take full responsibility for the final version of the work.

## Disclosures

A. Haeberlin has received travel fees/educational grants from Medtronic, Biotronik, Abbott, and Philips/Spectranetics without impact on his personal remuneration. He serves as a proctor for Medtronic. He has received research grants from the Swiss National Science Foundation, the Swiss Innovation Agency Innosuisse, the Swiss Heart Foundation, the University of Bern, the University Hospital Bern, the Velux Foundation, the Hasler Foundation, the Swiss Heart Rhythm Foundation, and the Novartis Research Foundation. He is the cofounder and chief executive officer of Act-Inno AG. L. Roten has received research grants from Medtronic, the Swiss National Foundation, the Swiss Heart Foundation, the Immanuel and Ilse Straub Foundation, and the Sitem-Insel Support Fund, all for work outside the submitted study. He has received speaker/consulting honoraria from Abbott and Medtronic. T. Reichlin has received research grants from the Swiss National Science Foundation, the Swiss Heart Foundation, the Sitem-Insel Support Fund, Biotronik, Boston Scientific, and Medtronic, all for work outside the submitted study. He has received speaker/consulting honoraria or travel support from Abbott/St. Jude Medical, Biosense Webster, Biotronik, Boston Scientific, and Medtronic. He has received support for his institution’s fellowship program from Abbott/St. Jude Medical, Biosense Webster, Biotronik, Boston Scientific, and Medtronic. N. Kozhuharov has received research grants from the Swiss National Science Foundation (P400PM-194477 and P5R5PM_210856), Gottfried und Julia Bangerter-Rhyner-Stiftung, Freiwillige Akademische Gesellschaft, L. & Th. La Roche Stiftung, the European Society of Cardiology, the European Union, and the Swiss Secretary of Education, Research and Innovation. F. Noti has received travel fees, speaker fees, and educational grants from Medtronic and Abbott; travel fees and educational grants from Boston Scientific and Philips Spectranetics; and an institutional grant from Biotronik, all for work outside the submitted study. T. Kueffer has received research grants from the Swiss Heart Foundation for work outside the submitted study. V. Spahiu has received travel fees and congress fees from Daichi Sankyo, Boston Scientific, and Johnson & Johnson. The authors have no conflicts of interest to disclose.

## References

[1] Leitz P., Wasmer K., Andresen C. (2022). The incidence, electrophysiological characteristics and ablation outcome of left atrial tachycardias after pulmonary vein isolation using three different ablation technologies. J Cardiovasc Dev Dis.

[2] Gerstenfeld E.P., Callans D.J., Dixit S. (2004). Mechanisms of organized left atrial tachycardias occurring after pulmonary vein isolation. Circulation.

[3] Akerström F., Bastani H., Insulander P., Schwieler J., Arias M.A., Jensen-Urstad M. (2014). Comparison of regular atrial tachycardia incidence after circumferential radiofrequency versus cryoballoon pulmonary vein isolation in real-life practice. J Cardiovasc Electrophysiol.

[4] Mikhaylov E.N., Bhagwandien R., Janse P.A., Theuns D.A., Szili-Torok T. (2013). Regular atrial tachycardias developing after cryoballoon pulmonary vein isolation: incidence, characteristics, and predictors. Europace.

[5] Wasmer K., Mönnig G., Bittner A. (2012). Incidence, characteristics, and outcome of left atrial tachycardias after circumferential antral ablation of atrial fibrillation. Heart Rhythm.

[6] Deisenhofer I., Estner H., Zrenner B. (2006). Left atrial tachycardia after circumferential pulmonary vein ablation for atrial fibrillation: incidence, electrophysiological characteristics, and results of radiofrequency ablation. Europace.

[7] Lyan E., Yalin K., Abdin A. (2019). Mechanism, underlying substrate and predictors of atrial tachycardia following atrial fibrillation ablation using the second-generation cryoballoon. J Cardiol.

[8] Yakabe D., Ohtani K., Araki M., Inoue S., Nakamura T. (2024). Long-term outcomes after catheter ablation for idiopathic atypical atrial flutter. Heart Rhythm.

[9] Zaidi A., Kirzner J., Liu C.F. (2024). Localized re-entry is a frequent mechanism of de novo atypical flutter. JACC Clin Electrophysiol.

[10] Servatius H., Kueffer T., Erdoes G. (2024). Electrophysiological differences of randomized deep sedation with dexmedetomidine versus propofol. BMC Anesthesiol.

[11] Servatius H., Küffer T., Baldinger S.H. (2022). Dexmedetomidine versus propofol for operator-directed nurse-administered procedural sedation during catheter ablation of atrial fibrillation: a randomized controlled study. Heart Rhythm.

[12] Kueffer T., Madaffari A., Thalmann G. (2023). Eliminating transseptal sheath exchange for pulsed field ablation procedures using a direct over-the-needle transseptal access with the Faradrive sheath. Europace.

[13] Kueffer T., Seiler J., Madaffari A. (2023). Pulsed-field ablation for the treatment of left atrial reentry tachycardia. J Interv Card Electrophysiol.

[14] Lam A., Küffer T., Hunziker L. (2021). Efficacy and safety of ethanol infusion into the vein of Marshall for mitral isthmus ablation. J Cardiovasc Electrophysiol.

[15] Li X., Li M., Zhang Y. (2023). Simplified stepwise anatomical ablation strategy for mitral isthmus: efficacy, efficiency, safety, and outcome. Europace.

[16] Zhu X., Chu H., Li J. (2021). New discovery of left atrial macroreentry tachycardia: originating from the spontaneous scarring of left atrial anterior wall. J Interv Cardiol.

[17] Kozhuharov N., Michou E., Wussler D. (2024). Quantifying hemodynamic cardiac stress and cardiomyocyte injury in normotensive and hypertensive acute heart failure. Biomedicines.

[18] Kariki O., Mililis P., Saplaouras A. (2025). Invasive management of atrial tachycardias using a novel lattice-tip catheter combining high-density mapping and dual ablation properties: initial real-world experience. J Interv Card Electrophysiol.

