# Supplementary material for: Characteristics, pathophysiological mechanisms, and ablation outcomes of patients with de novo left atrial flutter compared with patients with postablation left atrial flutter
Source: Heart Rhythm O2. 2026 Mar 27;7(7):1203–11. doi: 10.1016/j.hroo.2026.03.027 (PMC13390071; doi:10.1016/j.hroo.2026.03.027)
Supplement: Supplementary Tables 1-4 [file mmc1.docx]

Table S1. Prior Ablation Lesion Sets and Arrhythmia Circuit Mechanism in the post-ablation Group (N=115).

|  | **Anteroseptal Line** | | **Posterior Wall Line** | | **Roof Line** | | **Lateral Line (incl. block line)** | |
| --- | --- | --- | --- | --- | --- | --- | --- | --- |
| **Arrhythmia Mechanism** | **Absent (n=40)** | **Present (n=75)** | **Absent (n=67)** | **Present (n=48)** | **Absent (n=91)** | **Present (n=24)** | **Absent (n=98)** | **Present (n=17)** |
| **Anteroseptal Localized** | 1 (2%) | 21 (28%) | 14 (21%) | 8 (17%) | 19 (21%) | 3 (12%) | 21 (21%) | 1 (6%) |
| **Other Localized Reentry** | 3 (8%) | 1 (1%) | 3 (4%) | 1 (2%) | 3 (3%) | 1 (4%) | 2 (2%) | 2 (12%) |
| **Perimitral** | 18 (45%) | 32 (43%) | 34 (51%) | 16 (33%) | 40 (44%) | 10 (42%) | 40 (41%) | 10 (59%) |
| **Posterior Localized** | 8 (20%) | 1 (1%) | 1 (1%) | 8 (17%) | 8 (9%) | 1 (4%) | 9 (9%) | — |
| **Roof Dependent** | 10 (25%) | 15 (20%) | 13 (19%) | 12 (25%) | 18 (20%) | 7 (29%) | 21 (21%) | 4 (24%) |
| **Unmappable** | 0 (0%) | 5 (7%) | 2 (3%) | 3 (6%) | 3 (3%) | 2 (8%) | 5 (5%) | — |

|  |  |  |  | **De-novo** |  |  |  | |  | |  | | **Post-ablation** | |  | |  |
| --- | --- | --- | --- | --- | --- | --- | --- | --- | --- | --- | --- | --- | --- | --- | --- | --- | --- |
|  | **Anteroseptal Localized (n=22)** | **Other Localized Reentry (n=4)** | **Lateral (n=50)** | **Posterior Localized (n=9)** | **Roof Dependent (n=25)** | **Unmappable (n=5)** |  | **Anteroseptal Localized  (n=14)** | | **Lateral (n=10)** | | **Posterior Localized (n=1)** | | **Roof Dependent (n=5)** | | **Unmappable (n=2)** | |
| **Anteroseptal Scar** | 18 (55%) | 1 (25%) | 27 (41%) | 1 (6%) | 10 (28%) | 5 (50%) |  | 13 (72%) | | 7 (54%) | | — | | 4 (50%) | | 2 (40%) | |
| **Posterior Wall Scar** | 7 (21%) | -- | 10 (15%) | 8 (47%) | 10 (28%) | 3 (30%) |  | 4 (22%) | | 3 (23%) | | 1 (100%) | | 3 (38%) | | 2 (40%) | |
| **Roof Scar** | 3 (9%) | — | 4 (6%) | — | 3 (8%) | 2 (20%) |  | 1 (6%) | | — | | — | | — | | 1 (20%) | |
| **Lateral Scar (incl. Mitral isthmus)** | 1 (3%) | 2 (50%) | 8 (12%) | 7 (41%) | 5 (14%) | — |  | — | | 1 (8%) | | — | | — | | — | |
| **No Scar** | 1 (3%) | — | 6 (9%) | — | 1 (3%) | — |  | — | | 2 (15%) | | — | | — | | — | |
| **Not Described** | 3 (9%) | 1 (25%) | 11 (17%) | 1 (6%) | 7 (19%) | — |  | — | | — | | — | | 1 (12%) | | — | |
| **Total scar location** | 33 | 6 | 68 | 17 | 36 | 10 |  | 18 | | 13 | | 1 | | 8 | | 5 | |

Table S2. Voltage abnormalities and prior ablation lesion sets according to arrhythmia mechanism in de-novo patients (left panel) and post-ablation patients (right panel). The denominator is the total number of scar observations within each arrhythmia mechanism column; patients may have scar in multiple locations.

Table S3. Voltage abnormalities (scar) and arrhythmia mechanism in the whole cohort

|  | **Anteroseptal  Localized** | **Other Localized  Reentry** | **Lateral** | **Posterior Localized** | **Roof Dependent** | **Unmappable** |
| --- | --- | --- | --- | --- | --- | --- |
| **Anteroseptal Scar** | 31 (60%) | 1 (25%) | 34 (43%) | 1 (5%) | 14 (32%) | 7 (47%) |
| **Posterior Wall Scar** | 11 (21%) | -- | 13 (19%) | 9 (47%) | 13 (30%) | 5 (33%) |
| **Roof Scar** | 4 (7%) | — | 4 (5%) | — | 3 (7%) | 3 (20%) |
| **Lateral Scar** | 2 (4%) | 2 (50%) | 7 (9%) | 8 (42%) | 5 (11%) | — |
| **No Scar** | 1 (2%) | — | 8 (10%) | — | 1 (2%) | — |
| **Not Described** | 3 (6%) | 1 (25%) | 11 (14%) | 1 (5%) | 8 (18%) | — |
| **Total scar observations (n)** | **52** | **4** | **77** | **19** | **44** | **15** |

The denominator is scar (low voltage areas), the total number of scars is higher than the number of arrhythmia circuits as patients may have scar in more than one location.

Table S4. Arrhythmia mechanism in 42 patients with recurrences and the scar localization during redo procedure.

| **Arrhythmia Mechanism** | **Anteroseptal**  **scar**  **(N=27)** | **Posterior**  **wall scar**  **(N=15)** | **Roof**  **scar**  **(N=6)** | **Lateral**  **scar**  **(N=5)** | **No scar**  **(N=3)** | **Not described**  **(N=5)** | **Total** |
| --- | --- | --- | --- | --- | --- | --- | --- |
| **Anteroseptal reentry** | 2 (7.4%) | 2 (13.3%) | 0 | 1 (20.0%) | 0 | 0 | 5 (11.9%) |
| **Posterior localized** | 5 (18.5%) | 3 (20.0%) | 1 (16.7%) | 0 | 0 | 0 | 9 (21.4%) |
| **Other localized** | 0 | 0 | 0 | 0 | 0 | 0 | 0 |
| **Perimitral** | 5 (18.5%) | 2 (13.3%) | 1 (16.7%) | 0 | 1 (33.3%) | 2 (40.0%) | 11 (26.2%) |
| **Roof-dependent** | 1 (3.7%) | 1(6.7%) | 0 | 0 | 1 (33.3%) | 1 (20.0%) | 4 (9.5%) |
| **Biatrial** | 1 (3.7%) | 0 | 0 | 0 | 0 | 0 | 1 (2.4%) |
| **Atrial fibrillation** | 6 (22.2%) | 5 (33.3%) | 3 (50.0%) | 3 (60.0%) | 1 (33.3%) | 1 (20.0%) | 19 (45.2%) |
| **Unmappable** | 7 (25.9%) | 2 (13.3%) | 1 (16.7%) | 1 (20.0%) | 0 | 1 (20.0%) | 12 (28.6%) |

**Supplemental Figure 1: Recurrence of atrial arrhythmias after ablation of LAF (RFA subgroup)**

Kaplan-Meier curve displaying freedom from any atrial arrhythmia after LAF ablation for the subgroup of patients treated with RFA. Comparison between patients with de-novo LAF and those with post-ablation LAF.

**Supplemental Figure 2: Recurrence of atrial arrhythmias after ablation of LAF (PFA subgroup)**

Kaplan-Meier curve displaying freedom from any atrial arrhythmia after LAF ablation for the subgroup of patients treated with PFA. Comparison between patients with de-novo LAF and those with post-ablation LAF.
