# Supplementary figures and images for: Characteristics, pathophysiological mechanisms, and ablation outcomes of patients with de novo left atrial flutter compared with patients with postablation left atrial flutter
Source: Heart Rhythm O2. 2026 Mar 27;7(7):1203–11. doi: 10.1016/j.hroo.2026.03.027 (PMC13390071; doi:10.1016/j.hroo.2026.03.027)

# LA Flutter ablation – RFA only

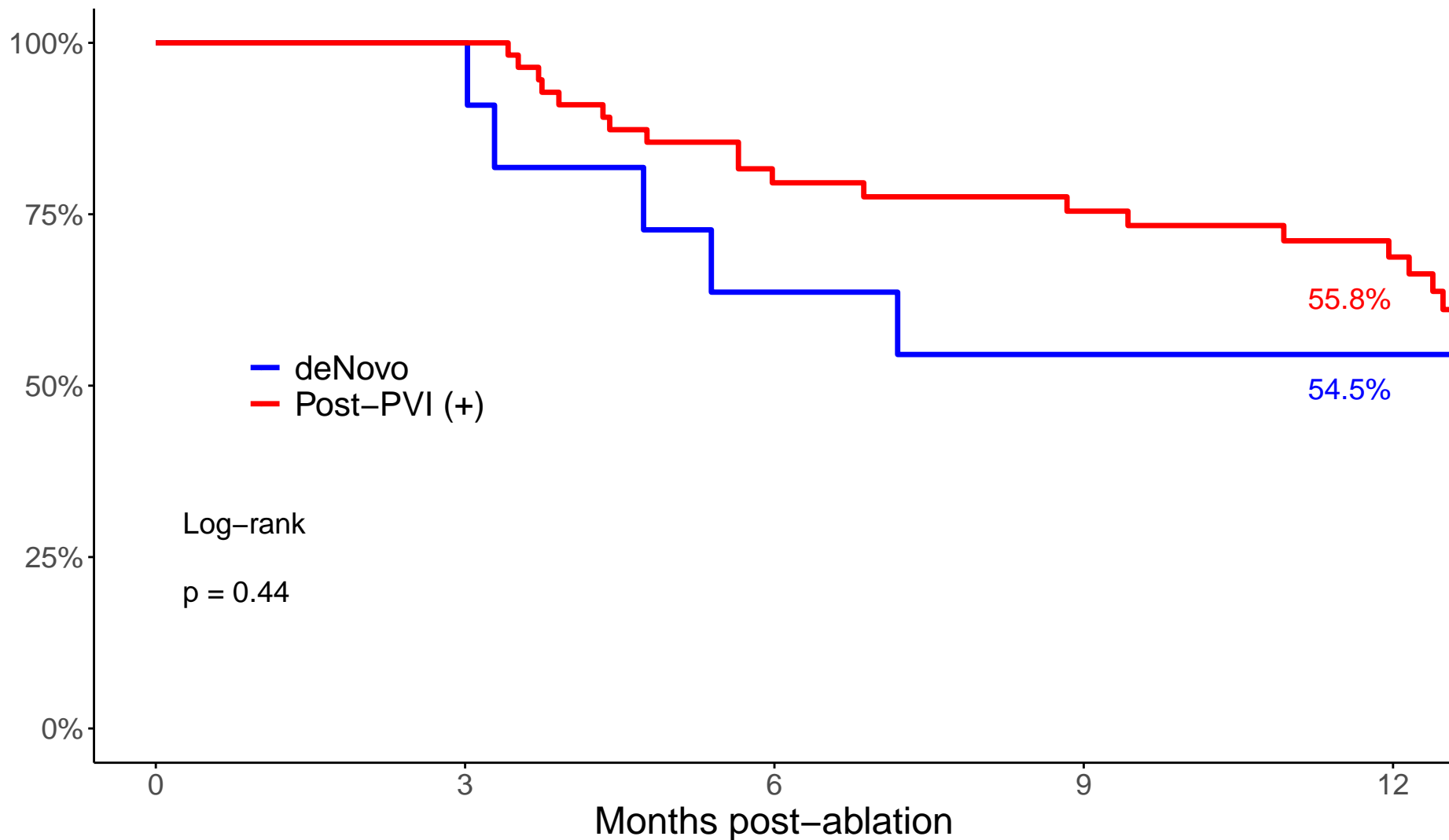

Number at risk: n (%)

|              |          |          |         |         |         |
|--------------|----------|----------|---------|---------|---------|
| deNovo       | 11 (100) | 11 (100) | 7 (64)  | 5 (45)  | 4 (36)  |
| Post-PVI (+) | 63 (100) | 58 (92)  | 39 (62) | 36 (57) | 29 (46) |
|              | 0        | 3        | 6       | 9       | 12      |

Months post-ablation

Supplement: Supplementary Figure 1 [file mmc2.pdf]

# LA Flutter ablation – PFA only

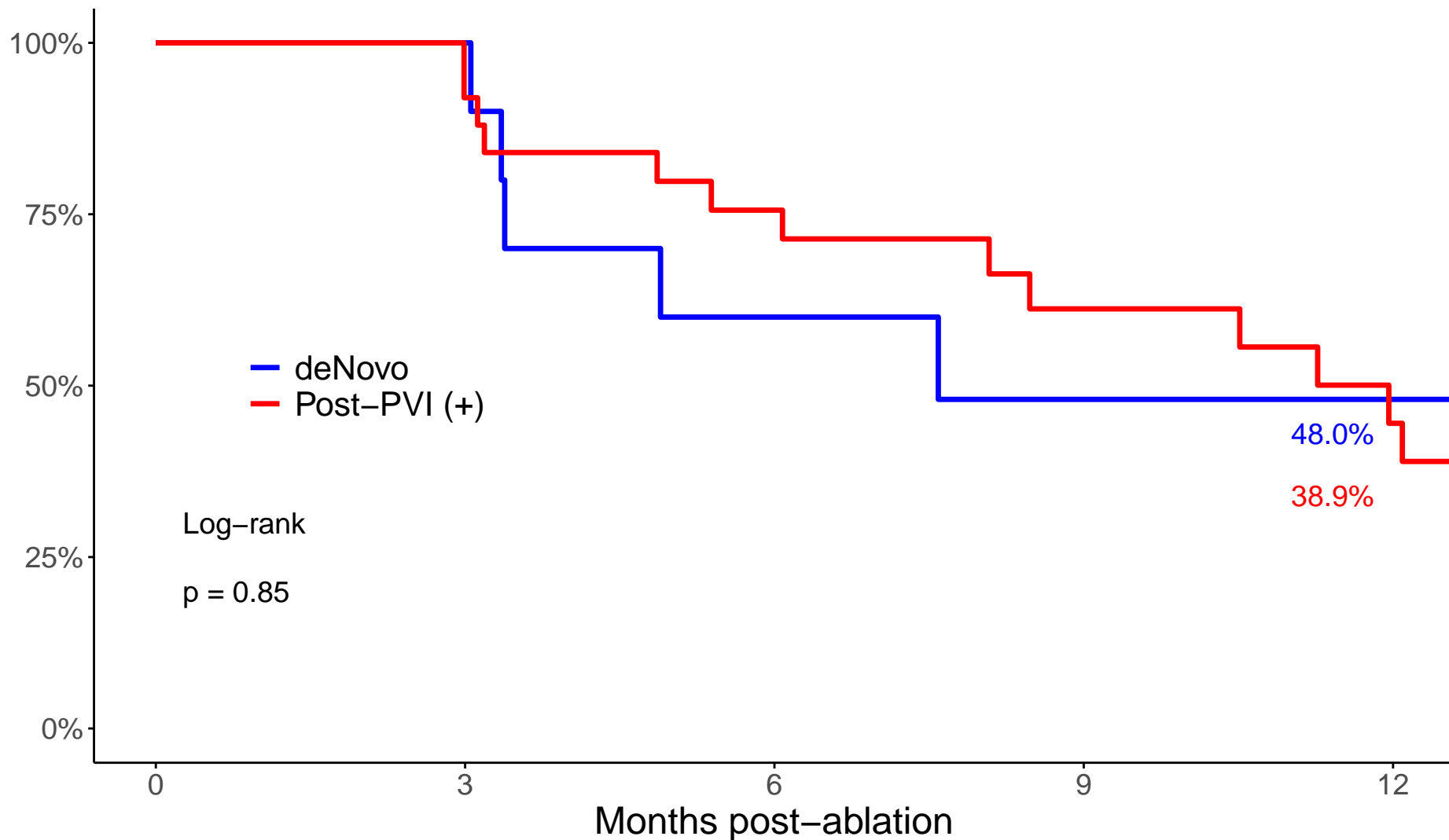

Number at risk: n (%)

|              |          |         |         |         |        |
|--------------|----------|---------|---------|---------|--------|
|              | 0        | 3       | 6       | 9       | 12     |
| deNovo       | 12 (100) | 10 (83) | 5 (42)  | 3 (25)  | 3 (25) |
| Post-PVI (+) | 27 (100) | 23 (85) | 18 (67) | 12 (44) | 8 (30) |

Months post-ablation

Supplement: Supplementary Figure 2 [file mmc3.pdf]
